# Multivisceral transplantation of pelvic organs in rats

**DOI:** 10.3389/fsurg.2023.1086651

**Published:** 2023-04-20

**Authors:** Flavio Henrique Ferreira Galvao, Jun Araki, Ana Bruna Salles Fonseca, Ruy Jorge Cruz, Cinthia Lanchotte, Daniel Reis Waisberg, Eleazar Chaib, Lucas Souto Nacif, Maria Clara de Camargo Traldi, Estrella Bianco de Mello, Wellington Andraus, Luiz Carneiro-D'Albuquerque

**Affiliations:** ^1^ Laboratory of Medical Investigation 37, Department of Gastroenterology, Hospital das Clínicas da Faculdade de Medicina da Universidade de São Paulo, São Paulo, Brazil; ^2^ Division of Plastic and Reconstructive Surgery, Shizuoka Cancer Center Hospital, Shizuoka, Japan; ^3^ Department of Surgery, University of Pittsburgh, Pittsburgh, PA, United States

**Keywords:** pelvic floor disorders, fecal incontinence, urinary incontinence, intestinal transplantation, tissue transplantation, anal canal, gender reassignment, dysphoria

## Abstract

**Background:**

Multivisceral transplantation of pelvic organs would be a potential treatment for severe pelvic floor dysfunction with fecal and urinary incontinence, extensive perineal trauma, or congenital disorders. Here, we describe the microsurgical technique of multivisceral transplantation of pelvic organs, including the pelvic floor, in rats.

**Donor operation:**

We performed a perineal (including the genitalia, anus, muscles, and ligaments) and abdominal incision. The dissection progressed near the pelvic ring, dividing ligaments, muscles, external iliac vessels, and pudendal nerves, allowing pelvic floor mobilization. The aorta and vena cava were isolated distally, preserving the internal iliac and gonadal vessels. The graft containing the skin, muscles, ligaments, bladder, ureter, rectum, anus and vagina, uterus and ovarian (female), or penile, testis and its ducts (male) was removed *en bloc*, flushed, and cold-stored.

**Recipient operation:**

The infrarenal aorta and vena cava were isolated and donor/recipient aorta-aorta and cava-cava end-to-side microanastomoses were performed. After pelvic floor and viscera removal, we performed microanastomoses between the donor and the recipient ureter, and the rectum and pudenda nerves. The pelvic floor was repositioned in its original position (orthotopic model) or the abdominal wall (heterotopic model). We sacrificed the animals 2 h after surgery.

**Results:**

We performed seven orthotopic and four heterotopic transplantations. One animal from the orthotopic model and one from the heterotopic model died because of technical failure. Six orthotopic and three heterotopic recipients survived up to 2 h after transplantation.

**Conclusion:**

The microsurgical technique for pelvic floor transplantation in rats is feasible, achieving an early survival rate of 81.82%.

## 1. Introduction

Multivisceral transplantation of pelvic organs would be a potential treatment for complex pelvic fecal and urinary incontinence. This condition is also called “dual incontinence” (DI) and remains a critical complication of pelvic floor dysfunction. The prevalence rate in the general population ranges between 5.3% and 9.4%, and in women, it increases to 20%–30% (1–5). Patients with complex DI frequently experience feelings of shame, social isolation, silent desperation, and serious impairment in their quality of life (1–5). Furthermore, there is no effective treatment for severe DI (6–9). Another potential indication for multivisceral transplantation of pelvic organs would be complex perineal defects secondary to congenital disorders or those caused by extensive trauma or burn.

Vascularized composite tissue allotransplantation—covering the face, members (arm, leg, hand, etc.), trachea, and anorectal segment, among others—is a recent advancement in the field of transplantation. This innovation aims to improve the quality of life and individual function, rather than mere survival, defining a new trend for the treatment of many system dysfunctions (10–13).

We hypothesize that pelvic floor transplantation covering the skin, pelvic muscular complex, urethra, bladder, ureter, genitalia (vagina or penile), anorectal segment, neurovascular pedicle, and secondary genital organs is a potential treatment for severe DI and complex perineal congenital disorders or injuries following extensive trauma. We have already observed in another study that the surgical technique for pelvic floor transplantation in cadavers is feasible (14). In this report, we describe the surgical technique and anatomic details of pelvic floor transplantation in rats, aiming at translational research.

## 2. Method

### 2.1 Animal and anesthesia procedures

Twenty-two Lewis rats weighing 250–300 g were used as donors and recipients in 11 pelvic floor transplantations. All procedures were approved by the research ethics committee of the University of São Paulo School of Medicine and the anesthesia was performed intraperitoneally using ketamine (30 mg/kg) and xylazine (10 ml/kg).

### 2.2 Surgical procedure

#### 2.2.1 Donor operation

**Figure 1:**
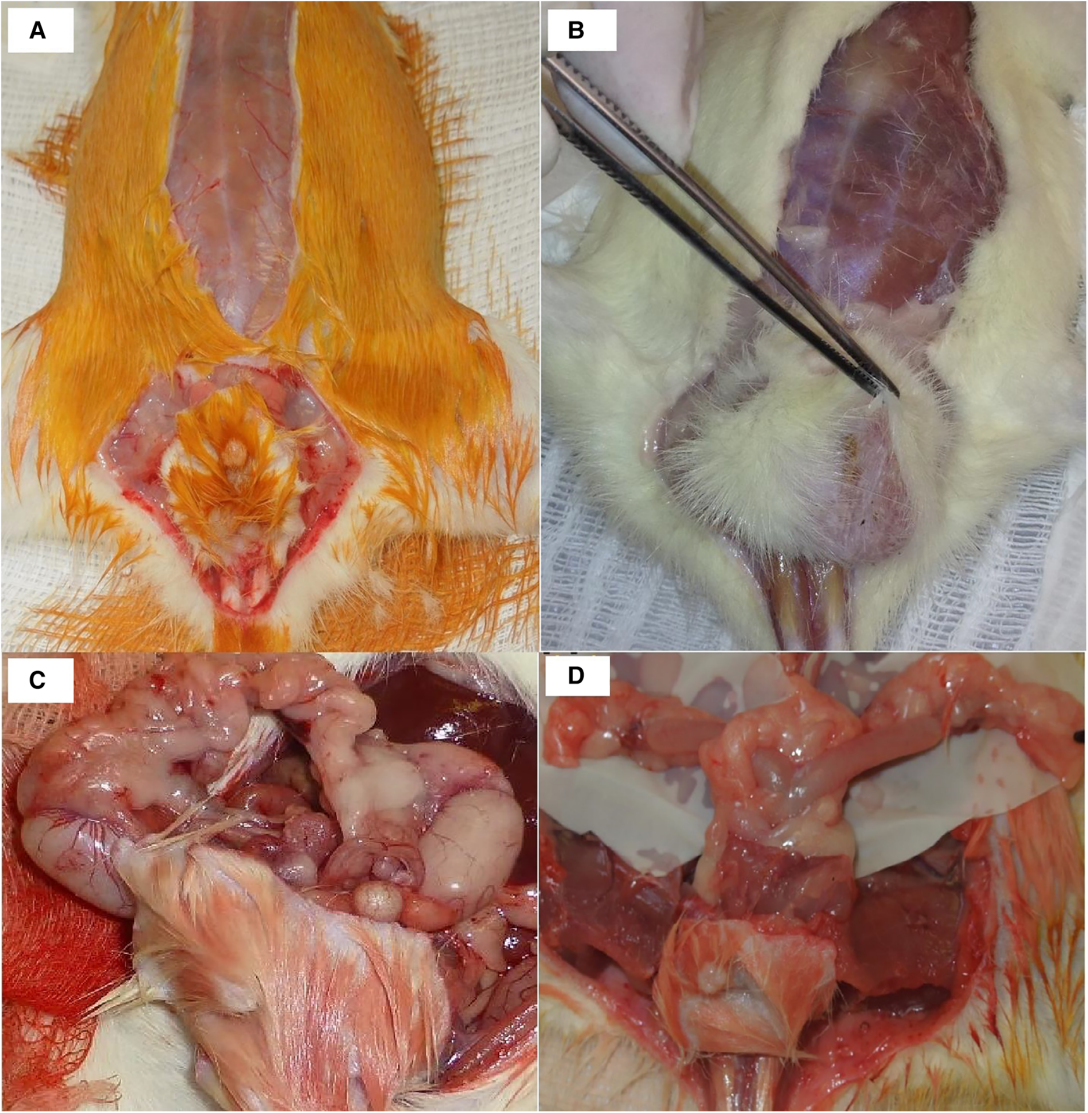
Incision details in male (**A**) and female (**B**) donors. Graft dissection in male (**C**) and female (**D**) rats.

**Figure 2:**
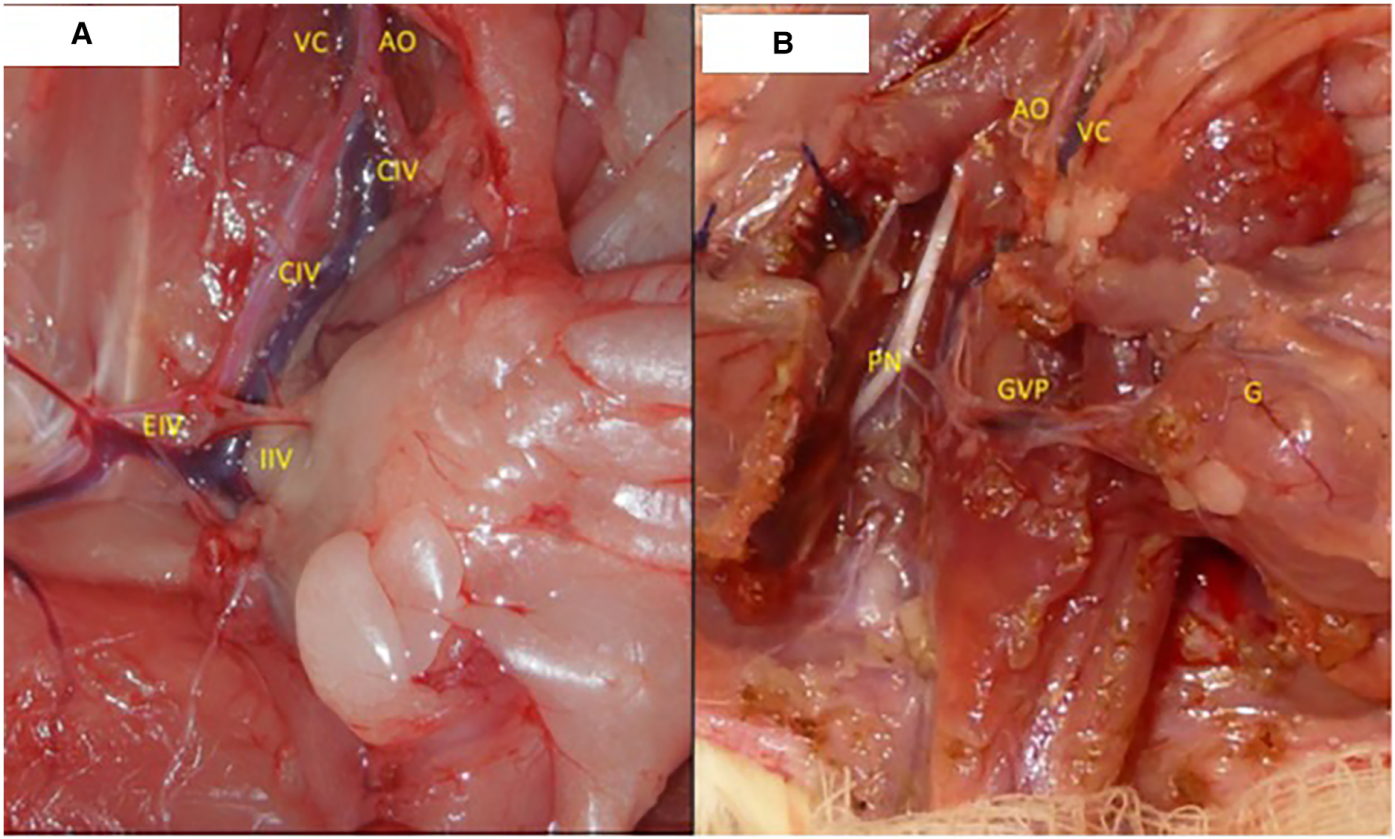
(**A**) Vascular dissection details: AO, aorta; VC, vena cava; IIV, internal iliac vessels; EIV, external iliac vessels; CIV, common iliac vessels. (**B**) Pedicle vessels from the final dissection and the source of the pudendal nerves: AO, aorta; VC, vena cava; NP, pudendal nerves; GVP, graft vascular pedicle; G, graft.

**Figure 3:**
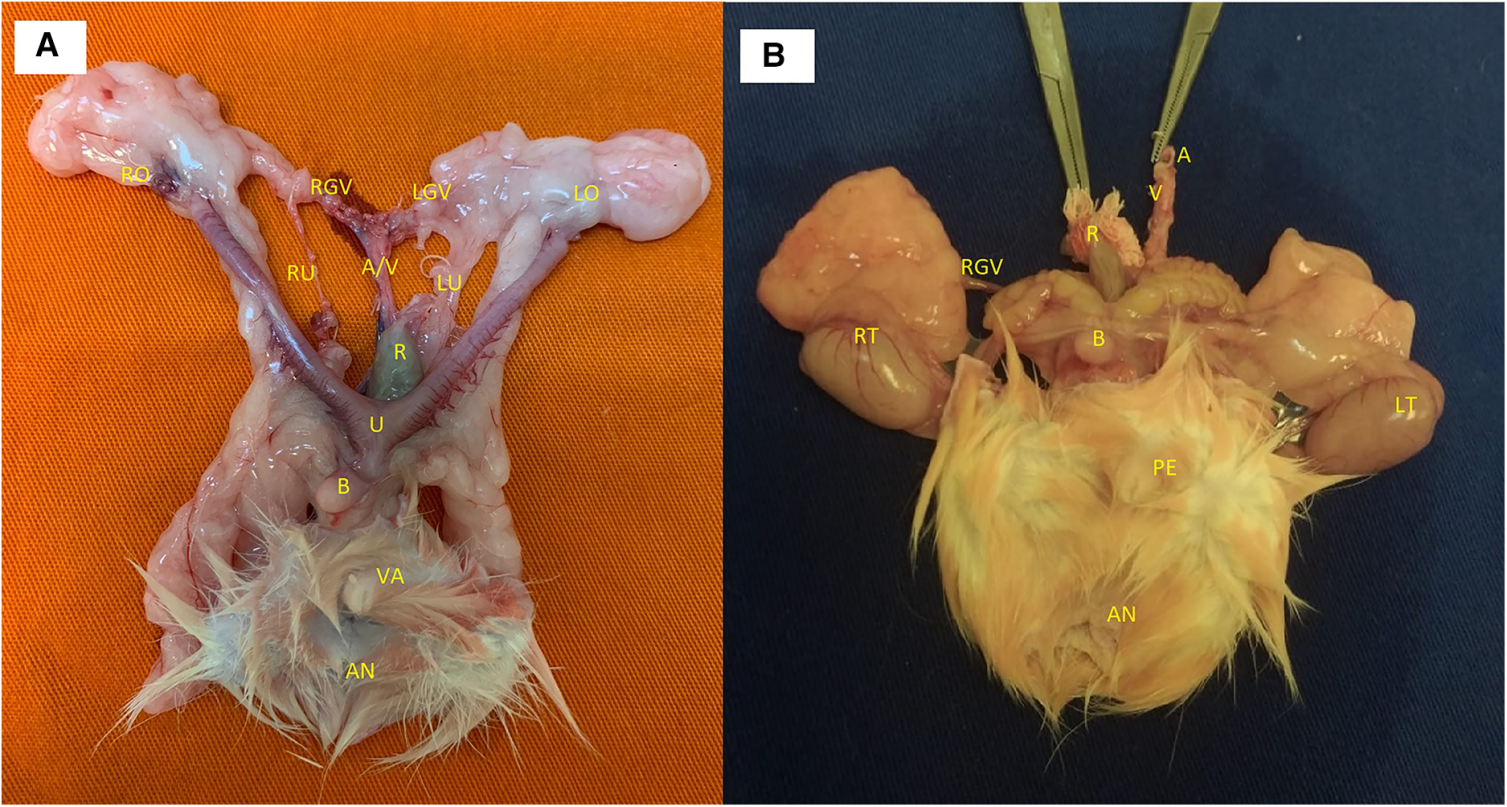
(**A**) female graft: RO, right ovary; LO,  left ovary; RGV,  right gonadal vessels; LGV, left gonadal vessels; A/V,  aorta and vena Cava; RU,  right ureter; LU,  left ureter; R, rectum; U,  uterus; B,  bladder; VA, vagina; AN, anus. (**B**) Male graft: RT, right testicle; LT, left testicle; RGV, right gonadal vessels; R, rectum; A, aorta; V,  vena cava; B, bladder; PE, penis; AN, anus.

A combined perineal and abdominal incision was performed (Figures 1A,B). The dissection progressed externally between the pelvic floor and the structures of the legs, abdominal wall, and gluteus and internally near the pelvic ring bone, preserving the perineal muscles, anorectal segment, and genitourinary organs of male (Figure 1C) and female (Figure 1D) donors. The division of the pubis makes this dissection easier and facilitates the identification of the vascular and pudendal nerves. The entire pelvic floor was mobilized to inside the abdomen. The genital organs, urinary bladder, and rectum were mobilized by performing the abdominal incision. The abdominal aorta and vena cava were isolated up to the renal vessels and down to the iliac bifurcation, preserving the internal iliac vessels, including the rectal vessels (Figure 2A). The pudendal nerves and vessels were identified and divided far from the pelvis (Figure 2B). The aorta and vena cava were sectioned near the renal vessels to preserve the gonadal vessels. The rectum was sectioned 3 cm before the anus and the ureters were sectioned 2 cm proximally from the bladder. Finally, we removed the graft *en bloc*, containing the skin, muscular complex, ligaments, bladder, ureter, rectum, anus and vagina, uterus and ovarian in female (Figure 3A) or penile, and testis and its ducts in male (Figure 3B) and placed it in a recipient containing a cold lactate Ringer solution. Immediately after, a catheter was inserted in the aorta to flush the graft with the Ringer solution. Afterward, the back-table procedures were performed.

#### 2.2.2 Recipient operation

**Figure 4:**
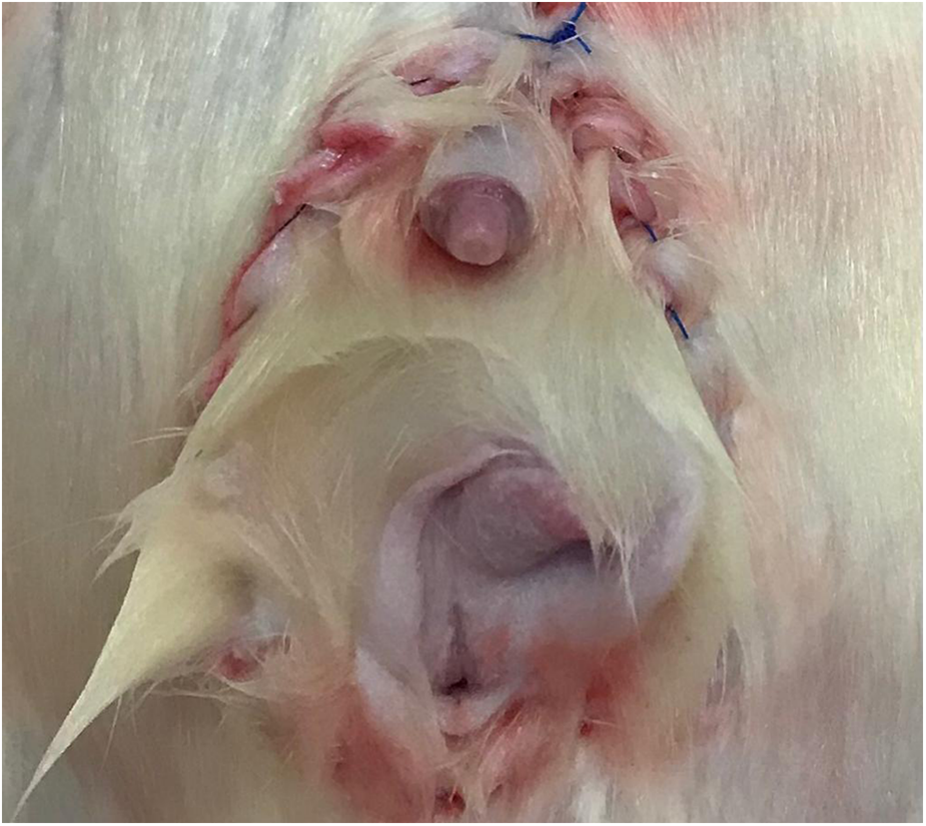
The final appearance of pelvic floor transplantation in a male subject.

We performed the same donor's anesthesia and abdominal incision and isolated a segment of 2 cm of the infrarenal aorta and vena cava for the anastomoses. We positioned the graft in the abdomen and performed end-to-side aorta-aorta and cava-cava microanastomoses using a 10.0 nylon suture. We removed the vascular clamps from the recipient's aorta and vena cava, allowing graft reperfusion. After that, we performed a similar perineal incision as that of the donor, and the internal iliac vessels were divided near the external vessels. We divided the pudendal nerves far from the sacrum, removed the native pelvic floor tissues, and performed end-to-end continuous anastomosis between the donor and the recipient rectum (7.0 polypropylene) and the pudendal nerves (10.0 nylon). For the ureter anastomosis, we used a polyethylene stent with a minimum ID of 0.5 mm inserted in both (donor and recipient) ureter ends and secured with two 7/0 silk ligatures. The pelvic floor was positioned in its original position (orthotopic) or in the lower part of the abdominal incision (heterotopic) and fixed by stitches between pelvic floor ligaments, muscles, and skin, completing the operation (Figure 4). We sacrificed the animals 2 h after surgery and removed the graft for histological analysis. The tissues were stained with hematoxylin and eosin for ischemia/reperfusion injury graduation.

## 3. Results

We performed a total of 11 consecutive pelvic floor transplantations in rats. Seven recipients were transplanted using the orthotopic model, whereas four were done with the heterotopic model. The reproductive organs were maintained in the graft, so the vascular pedicle included the gonadal vessels for their vascularization. Consequently, a long venous and arterial pedicle including the renal vessels was required. The donor/recipient size match is very important (4). Donor/recipient weight should be the same or the donor's weight should be slightly smaller to prevent compartmental syndrome. Furthermore, in this technique, we access the axis of the vascular (internal iliac vessels) and neuronal (pudendal nerves) pedicles, which allows larger vessels for anastomosis and provides higher chances of regeneration and functional reconstitution. The mean time for the donor's operation was 62.54 ± 11.45 min. For the recipient's operation, it was 108.14 ± 16.32 min for the orthotopic model and 68 ± 10.89 min for the heterotopic one. All grafts achieved normal color and good arterial pulse after reperfusion, and nine animals survived up to 2 h after the surgical procedure (experiment endpoint), with six in the orthotopic group and three in the heterotopic group. Two animals died before this period, one because of bleeding from perineal dissection (orthotopic fashion) and the other because of bleeding from arterial microanastomosis (heterotopic fashion). The histology assessment of the present investigation (normal = 0, mild = I, moderate = II, and severe = III) was based on the anorectal transplantation histological classification that searched for ischemic/reperfusion injury (mainly inflammatory cell infiltration and edema) in all organs of the composite tissue transplanted (15, 16). In the current research, ischemia/reperfusion injury was graded 0 in five grafts and 1 in four grafts. The small amount of lesion in the present investigation was probably due to the short period of cold ischemia. All animals that survived during the heterotopic model procedure recovered from anesthesia until the experiment endpoint, probably because of the shorter surgical time. In the orthotopic model, the three animals with the shortest surgical time also fully recovered from anesthesia. The heterotopic model of pelvic floor transplantation was conceptualized to develop a two-step technique in which the graft is implanted heterotopically first and then, after 2 days, another surgery is performed to remove the recipient's pelvic organs and to place the graft in orthotopic position. We believe that this two-step technique may improve the survival rate of patients undergoing this complex procedure.

## 4. Discussion

Our group and others have investigated anorectal transplantation as a potential solution for severe fecal incontinence. The initial results of this procedure in rats, swine, and canines are promising, showing a convenient functional recovery of this composite graft transplantation. Thus, these experiments suggest that anorectal transplantation would be a promising solution for severe fecal incontinence and permanent colostomy (15–24).

The necessity of anatomic studies for our research in anorectal transplantation inspired us to develop pelvic floor transplantation. During pelvic floor dissection in cadavers and rats, we observed that the pelvic floor is a complex system working like a singular organ. They share the same vascular and neuronal pedicles that control the musculature, which promotes both urinary and fecal continence as well as sexual reproductive functions. Thus, we formulated the hypothesis that the entire structure could be transplanted as a composite graft.

Araki et al. suggest that the anastomosis of pudendal vessels by super-microsurgery would be important for the recovery of pudendal function after anal transplantation, affecting all pelvic organs (22, 23). Nevertheless, in a recent series of anorectal transplantations in rats, we observed adequate anorectal recovery without pudendal vessel anastomosis (16). Furthermore, we could observe satisfactory anal function in a heterotopic model of anal autotransplantation (19), suggesting a profound influence of intrinsic bowel innervation on the rat's anorectal function. These thought-provoking results demand further research studies for easy elucidation.

Many patients would benefit from this procedure, including those with complex perineal trauma and congenital pelvic deformation. We are currently designing new models to expand the indications of this composite graft, mainly for trauma. Another possible indication would be for sexual gender reassignment for patients with gender identity disorder (dysphoria). Gender reassignment surgeries have been indicated for these patients; however, these procedures create artificial organs and may cause intricate complications (25, 26). Pelvic floor transplantation research may explore new possibilities depending on the demand for the inclusion of cross-gender pelvic floor transplantation with the reproductive organs for dysphoria or inclusion in the graft of parts of the pelvic bone framework, gluteus, abdominal wall, and limbs, with their respective vascular and nervous pedicles, for complex trauma or congenital disorders.

Currently, we are designing in our laboratory new experiments to observe the long-term survival rates of rats in pelvic floor transplantation as well as the function of the graft. This is a highly complex procedure that requires high microsurgical skills and intensive peri- and postoperative care, including hemodynamic and biochemical monitoring, respiratory support, administration of antibiotics, fluid infusion, and potential blood transfusion.

The possibility of the genitalia and adnexa transfer in the present procedure may increase the bioethical and metaphysical concerns of patients; nevertheless, these anxieties would be similar to those that exist in gender reassignment surgeries, which are already well accepted as a current therapeutic option for gender identity disorder (25, 26). Furthermore, additional refined, basic, and preclinic translational research of this procedure and an intense and reflective community debate about this medical advancement would be necessary to prepare society for this innovation, mainly pertaining to the donation of these composite tissues by the deceased. Another concern would be the high amount of immunogenic tissues present in this graft like skin, which would importantly require permanent immunosuppression after transplantation. The current procedure the current procedure would request similar immunosuppression used for the face, arms, and lower-extremity transplantation (10, 12, 13), which also enclose high immunogenic graft and need treatment for rejection by specific immunosuppressive induction therapy and maintenance immunosuppression, mainly with tacrolimus.

Pelvic floor transplantation may be a relevant option for severe pelvic floor dysfunction caused by extensive trauma or complex congenital disorders. This procedure is feasible in rats and opens the door for meaningful ethical, biotechnological, anatomical, and surgical debate.

## Data Availability

The original contributions presented in the study are included in the article/Supplementary Material; further inquiries can be directed to the corresponding author.
